# *trans*-2-(2-Nitro-1-phenyl­eth­yl)cyclo­hexa­none

**DOI:** 10.1107/S1600536810045423

**Published:** 2010-11-13

**Authors:** Ivo Zenz, Herbert Mayr, Peter Mayer

**Affiliations:** a Ludwig-Maximilians-Universität, Department, Butenandtstrasse 5–13, 81377 München, Germany

## Abstract

In the title compound, C_14_H_17_NO_3_, the plane of the phenyl ring and the least-squares plane of the cyclo­hexyl moiety enclose an angle of 89.14 (6)°. The cyclohexyl ring adopts a chair conformation. In the crystal, the molecules are linked by weak C—H⋯O bonds, with each of the nitro-O atoms accepting two such interactions.

## Related literature

For the history and synthesis of nitro­alkenes, see: Tsogoeva *et al.* (2007 ▶); Sulzer-Mosse & Alexakis (2007 ▶); Mukherjee *et al.* (2007 ▶); Kempf *et al.* (2003 ▶); Blarer *et al.* (1982 ▶); Juaristi *et al.* (1993 ▶). For related structures, see: Cobb *et al.* (2005 ▶), Xu *et al.* (2007*a* ▶,*b* ▶).
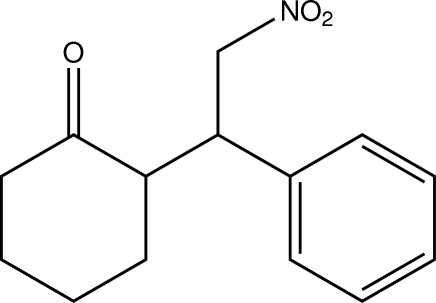


## Experimental

### 

#### Crystal data

C_14_H_17_NO_3_
*M* *_r_* = 247.29
Monoclinic, 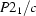

*a* = 13.4567 (6) Å
*b* = 8.3618 (4) Å
*c* = 11.3668 (5) Å
β = 91.734 (4)°
*V* = 1278.43 (10) Å^3^
*Z* = 4
Mo *K*α radiation
μ = 0.09 mm^−1^
*T* = 173 K
0.38 × 0.27 × 0.18 mm

#### Data collection

Oxford Xcalibur diffractometer
Absorption correction: multi-scan (*CrysAlis PRO*; Oxford Diffraction, 2006 ▶) *T*_min_ = 0.986, *T*_max_ = 1.000
9360 measured reflections
2605 independent reflections
1829 reflections with *I* > 2σ(*I*)
*R*_int_ = 0.026

#### Refinement

*R*[*F* ^2^ > 2σ(*F* ^2^)] = 0.036
*wR*(*F* ^2^) = 0.090
*S* = 0.98
2605 reflections
163 parameters
H-atom parameters constrained
Δρ_max_ = 0.17 e Å^−3^
Δρ_min_ = −0.17 e Å^−3^

### 

Data collection: *CrysAlis PRO* (Oxford Diffraction, 2006 ▶); cell refinement: *CrysAlis PRO*; data reduction: *CrysAlis PRO*; program(s) used to solve structure: *SIR97* (Altomare *et al.*, 1999 ▶); program(s) used to refine structure: *SHELXL97* (Sheldrick, 2008 ▶); molecular graphics: *ORTEP-3 for Windows* (Farrugia, 1997 ▶) and *Mercury* (Macrae *et al.*, 2006 ▶); software used to prepare material for publication: *PLATON* (Spek, 2009 ▶).

## Supplementary Material

Crystal structure: contains datablocks I, global. DOI: 10.1107/S1600536810045423/ng5052sup1.cif

Structure factors: contains datablocks I. DOI: 10.1107/S1600536810045423/ng5052Isup2.hkl

Additional supplementary materials:  crystallographic information; 3D view; checkCIF report

## Figures and Tables

**Table 1:** Hydrogen-bond geometry (Å, °)

*D*—H⋯*A*	*D*—H	H⋯*A*	*D*⋯*A*	*D*—H⋯*A*
C1—H1*B*⋯O2^i^	0.99	2.57	3.4403 (14)	146
C5—H5*A*⋯O2^ii^	0.99	2.47	3.4312 (16)	165
C8—H8*A*⋯O1^iii^	0.99	2.53	3.3536 (16)	140
C10—H10⋯O2^i^	0.95	2.50	3.4289 (15)	165

Symmetry codes: (i) 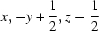; (ii) 
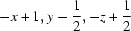
; (iii) 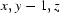.

**Fig. 1.**
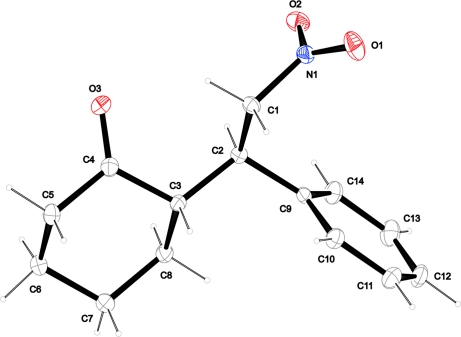
The molecular structure of the title compound, with atom labels and anisotropic displacement ellipsoids (drawn at 50% probability level) for non-H atoms.

**Fig. 2.**
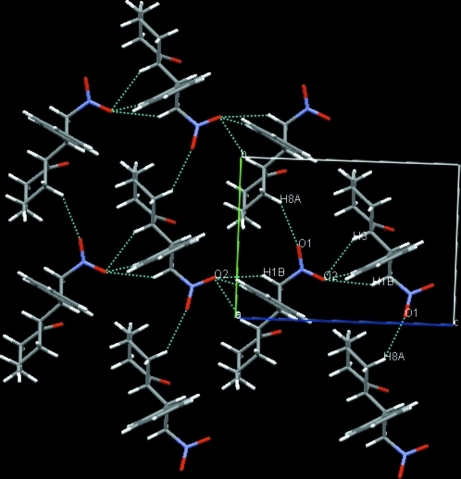
Weak intermolecular interactions in the crystal structure of the title compound viewed along [100].

## supplementary crystallographic information

### Comment

Nitroalkenes are important reagents in organic chemistry and they are the most prominent Michael acceptors used in organocatalytic reactions [Tsogoeva *et al.* (2007), Sulzer-Mosse *et al.* (2007), Mukherjee *et al.* (2007)]. During our studies on the electrophilic reactivity of *trans-β*-nitrostyrenes, we employed enamines of known nucleophilic reactivities and, hence, obtained the title compound from a reaction of *trans-β*-nitrostyrene and 1-pyrrolidinocyclohexene [Kempf *et al.* (2003), Blarer *et al.* (1982), Juaristi *et al.* (1993)].

In the title compound, the 1'-Phenyl-2'-nitro-ethyl moiety occupies an equatorial binding site in 2-position of the cyclohexanone ring (see Fig. 1). The plane of the phenyl ring and the least-square plane of the cyclohexyl moiety enclose an angle of 89.14 (6)° which is close to the angles found in enantiopure crystals of the title compound (87.1 (3)° at 180 K (Cobb *et al.* (2005)), 87.40 (8)° at 296 K (Xu *et al.*, 2007a,b)). The plane through the nitro group and the adjacent C1 atom encloses an angle of 68.81 (7)° with the phenyl ring.

Taking into account merely interactions with hydrogen-acceptor distances at least 0.1 Å shorter than the sum of van-der-Waals radii, the molecules are linked by very weak contacts of the type C—H···O with nitro-O atoms as acceptors (see Fig. 2). The molecular structure of the title compound is stabilized by these contacts as well, as the involved hydrogen atoms are located in the cyclohexyl ring, the phenyl ring and the nitro-ethyl side chain. The keto group is not involved in hydrogen bonding. π-stacking and C—H···π-interactions are not observed.

### Experimental

*trans*-2-[1'-Phenyl-2'-nitro-ethyl]-cyclohexanone has been obtained by dissolving *trans-β*-nitrostyrene (4.05 mmol, 604 mg) in dry diethylether (40 ml) and dropwise addition of 1-pyrrolidinocyclohexene (4.05 mmol, 612 mg) at -78 °C. After stirring the reaction at RT for 2 h, 60 ml water, 60 ml e thanol and 5 ml 1*M* HCl have been added, and the mixture was stirred for 30 min at 60 °C. After removing the solvent *in vacuo*, a white solid has been obtained (3.01 mmol, 745 mg, 74%).

Crystallization procedure: The title compound was dissolved in ethanol and heated to the boiling point. The solvent was allowed to cool slowly to room temperature. After 24 h, colourless crystals had formed that were suitable for X-ray analysis; mp 108 °C.

### Refinement

All H atoms were calculated in ideal geometry and refined in a riding model.

### Crystal data

**Table:**

C_14_H_17_NO_3_	*F*(000) = 528
*M**_r_* = 247.29	*D*_x_ = 1.285 (1) Mg m^−^^3^
Monoclinic, *P*2_1_/*c*	Mo *K*α radiation, λ = 0.71073 Å
Hall symbol: -P 2ybc	Cell parameters from 3796 reflections
*a* = 13.4567 (6) Å	θ = 4.3–26.3°
*b* = 8.3618 (4) Å	µ = 0.09 mm^−^^1^
*c* = 11.3668 (5) Å	*T* = 173 K
β = 91.734 (4)°	Block, colourless
*V* = 1278.43 (10) Å^3^	0.38 × 0.27 × 0.18 mm
*Z* = 4	

### Data collection

**Table:**

Oxford Xcalibur diffractometer	2605 independent reflections
Radiation source: fine-focus sealed tube	1829 reflections with *I* > 2σ(*I*)
graphite	*R*_int_ = 0.026
ω scans	θ_max_ = 26.4°, θ_min_ = 4.3°
Absorption correction: multi-scan (*CrysAlis PRO*; Oxford Diffraction, 2006)	*h* = −16→16
*T*_min_ = 0.986, *T*_max_ = 1.000	*k* = −10→10
9360 measured reflections	*l* = −14→14

### Refinement

**Table:**

Refinement on *F*^2^	Primary atom site location: structure-invariant direct methods
Least-squares matrix: full	Secondary atom site location: difference Fourier map
*R*[*F*^2^ > 2σ(*F*^2^)] = 0.036	Hydrogen site location: inferred from neighbouring sites
*wR*(*F*^2^) = 0.090	H-atom parameters constrained
*S* = 0.98	*w* = 1/[σ^2^(*F*_o_^2^) + (0.0503*P*)^2^] where *P* = (*F*_o_^2^ + 2*F*_c_^2^)/3
2605 reflections	(Δ/σ)_max_ < 0.001
163 parameters	Δρ_max_ = 0.17 e Å^−^^3^
0 restraints	Δρ_min_ = −0.17 e Å^−^^3^

### Special details

**Table:**

Experimental. CrysAlisPro, Oxford Diffraction Ltd., Version 1.171.33.41 (release 06-05-2009 CrysAlis171 .NET) (compiled May 6 2009,17:20:42) Empirical absorption correction using spherical harmonics, implemented in SCALE3 ABSPACK scaling algorithm.

### Fractional atomic coordinates and isotropic or equivalent isotropic displacement parameters (Å^2^)

**Table:**

	*x*	*y*	*z*	*U*_iso_*/*U*_eq_	
O1	0.27791 (8)	0.45264 (12)	0.27671 (8)	0.0497 (3)	
O2	0.30532 (7)	0.25646 (11)	0.39600 (7)	0.0361 (3)	
O3	0.46549 (7)	−0.07785 (11)	0.20442 (8)	0.0378 (3)	
N1	0.30166 (8)	0.31433 (13)	0.29688 (9)	0.0272 (3)	
C1	0.32921 (9)	0.20990 (14)	0.19718 (10)	0.0243 (3)	
H1A	0.4012	0.1854	0.2029	0.029*	
H1B	0.3155	0.2661	0.1218	0.029*	
C2	0.26933 (9)	0.05426 (14)	0.20005 (10)	0.0214 (3)	
H2	0.2817	0.0036	0.2788	0.026*	
C3	0.30432 (9)	−0.06309 (14)	0.10615 (10)	0.0217 (3)	
H3	0.2931	−0.0124	0.0272	0.026*	
C4	0.41304 (9)	−0.11083 (15)	0.11829 (11)	0.0258 (3)	
C5	0.44795 (10)	−0.21687 (16)	0.02103 (11)	0.0333 (3)	
H5A	0.5197	−0.2403	0.0330	0.040*	
H5B	0.4383	−0.1623	−0.0558	0.040*	
C6	0.38803 (10)	−0.37313 (16)	0.02159 (11)	0.0333 (3)	
H6A	0.4068	−0.4406	−0.0458	0.040*	
H6B	0.4037	−0.4330	0.0950	0.040*	
C7	0.27722 (9)	−0.33748 (16)	0.01310 (11)	0.0325 (3)	
H7A	0.2397	−0.4386	0.0205	0.039*	
H7B	0.2605	−0.2914	−0.0653	0.039*	
C8	0.24528 (10)	−0.22137 (15)	0.10807 (11)	0.0279 (3)	
H8A	0.2551	−0.2724	0.1862	0.033*	
H8B	0.1735	−0.1978	0.0966	0.033*	
C9	0.15860 (9)	0.08735 (14)	0.18614 (10)	0.0220 (3)	
C10	0.11872 (9)	0.16173 (15)	0.08584 (10)	0.0298 (3)	
H10	0.1618	0.1982	0.0268	0.036*	
C11	0.01755 (10)	0.18327 (17)	0.07092 (12)	0.0358 (3)	
H11	−0.0082	0.2349	0.0021	0.043*	
C12	−0.04660 (10)	0.13068 (16)	0.15475 (12)	0.0368 (4)	
H12	−0.1163	0.1439	0.1434	0.044*	
C13	−0.00848 (11)	0.05889 (17)	0.25497 (13)	0.0393 (4)	
H13	−0.0521	0.0231	0.3136	0.047*	
C14	0.09322 (10)	0.03820 (16)	0.27118 (11)	0.0316 (3)	
H14	0.1186	−0.0103	0.3415	0.038*	

### Atomic displacement parameters (Å^2^)

**Table:**

	*U*^11^	*U*^22^	*U*^33^	*U*^12^	*U*^13^	*U*^23^
O1	0.0667 (8)	0.0264 (6)	0.0566 (7)	0.0132 (5)	0.0092 (5)	−0.0020 (5)
O2	0.0429 (6)	0.0418 (6)	0.0236 (5)	−0.0013 (5)	0.0033 (4)	−0.0039 (4)
O3	0.0260 (5)	0.0419 (6)	0.0449 (6)	0.0040 (4)	−0.0083 (4)	−0.0096 (5)
N1	0.0240 (6)	0.0270 (6)	0.0306 (6)	−0.0007 (5)	0.0020 (4)	−0.0046 (5)
C1	0.0260 (7)	0.0258 (7)	0.0212 (6)	−0.0004 (6)	0.0048 (5)	−0.0022 (5)
C2	0.0215 (6)	0.0223 (7)	0.0204 (6)	0.0001 (5)	0.0014 (5)	0.0015 (5)
C3	0.0202 (6)	0.0225 (7)	0.0224 (6)	0.0013 (5)	−0.0008 (5)	0.0007 (5)
C4	0.0233 (7)	0.0234 (7)	0.0308 (7)	−0.0014 (6)	0.0026 (5)	0.0010 (5)
C5	0.0247 (7)	0.0381 (8)	0.0373 (7)	0.0055 (6)	0.0055 (6)	−0.0061 (6)
C6	0.0321 (8)	0.0299 (8)	0.0377 (7)	0.0080 (6)	−0.0019 (6)	−0.0082 (6)
C7	0.0309 (8)	0.0269 (7)	0.0395 (8)	0.0019 (6)	−0.0040 (6)	−0.0070 (6)
C8	0.0215 (7)	0.0253 (7)	0.0368 (7)	−0.0004 (6)	0.0004 (5)	−0.0035 (6)
C9	0.0222 (7)	0.0183 (6)	0.0255 (6)	0.0010 (5)	0.0011 (5)	−0.0044 (5)
C10	0.0270 (7)	0.0326 (8)	0.0299 (7)	0.0017 (6)	0.0018 (5)	0.0017 (6)
C11	0.0315 (8)	0.0384 (8)	0.0371 (8)	0.0066 (7)	−0.0052 (6)	0.0032 (6)
C12	0.0202 (7)	0.0360 (8)	0.0540 (9)	0.0061 (6)	−0.0015 (6)	−0.0032 (7)
C13	0.0274 (8)	0.0412 (9)	0.0500 (8)	0.0009 (7)	0.0126 (6)	0.0048 (7)
C14	0.0277 (7)	0.0332 (8)	0.0340 (7)	0.0031 (6)	0.0053 (6)	0.0060 (6)

### Geometric parameters (Å, °)

**Table:**

O1—N1	1.2198 (13)	C6—H6A	0.9900
O2—N1	1.2258 (12)	C6—H6B	0.9900
O3—C4	1.2210 (14)	C7—C8	1.5234 (17)
N1—C1	1.4865 (15)	C7—H7A	0.9900
C1—C2	1.5316 (16)	C7—H7B	0.9900
C1—H1A	0.9900	C8—H8A	0.9900
C1—H1B	0.9900	C8—H8B	0.9900
C2—C9	1.5192 (17)	C9—C14	1.3888 (17)
C2—C3	1.5343 (16)	C9—C10	1.3919 (16)
C2—H2	1.0000	C10—C11	1.3786 (18)
C3—C4	1.5187 (17)	C10—H10	0.9500
C3—C8	1.5442 (16)	C11—C12	1.3770 (19)
C3—H3	1.0000	C11—H11	0.9500
C4—C5	1.5035 (18)	C12—C13	1.3732 (19)
C5—C6	1.5355 (19)	C12—H12	0.9500
C5—H5A	0.9900	C13—C14	1.3862 (19)
C5—H5B	0.9900	C13—H13	0.9500
C6—C7	1.5209 (18)	C14—H14	0.9500
			
O1—N1—O2	123.37 (10)	C7—C6—H6B	109.6
O1—N1—C1	118.92 (10)	C5—C6—H6B	109.6
O2—N1—C1	117.70 (10)	H6A—C6—H6B	108.1
N1—C1—C2	109.84 (9)	C6—C7—C8	112.15 (10)
N1—C1—H1A	109.7	C6—C7—H7A	109.2
C2—C1—H1A	109.7	C8—C7—H7A	109.2
N1—C1—H1B	109.7	C6—C7—H7B	109.2
C2—C1—H1B	109.7	C8—C7—H7B	109.2
H1A—C1—H1B	108.2	H7A—C7—H7B	107.9
C9—C2—C1	110.98 (10)	C7—C8—C3	112.33 (10)
C9—C2—C3	111.39 (9)	C7—C8—H8A	109.1
C1—C2—C3	110.85 (9)	C3—C8—H8A	109.1
C9—C2—H2	107.8	C7—C8—H8B	109.1
C1—C2—H2	107.8	C3—C8—H8B	109.1
C3—C2—H2	107.8	H8A—C8—H8B	107.9
C4—C3—C2	114.83 (9)	C14—C9—C10	117.75 (12)
C4—C3—C8	105.55 (10)	C14—C9—C2	120.91 (10)
C2—C3—C8	111.68 (10)	C10—C9—C2	121.29 (11)
C4—C3—H3	108.2	C11—C10—C9	120.93 (12)
C2—C3—H3	108.2	C11—C10—H10	119.5
C8—C3—H3	108.2	C9—C10—H10	119.5
O3—C4—C5	122.47 (12)	C12—C11—C10	120.70 (12)
O3—C4—C3	123.05 (11)	C12—C11—H11	119.7
C5—C4—C3	114.19 (11)	C10—C11—H11	119.7
C4—C5—C6	108.84 (11)	C13—C12—C11	119.17 (13)
C4—C5—H5A	109.9	C13—C12—H12	120.4
C6—C5—H5A	109.9	C11—C12—H12	120.4
C4—C5—H5B	109.9	C12—C13—C14	120.48 (13)
C6—C5—H5B	109.9	C12—C13—H13	119.8
H5A—C5—H5B	108.3	C14—C13—H13	119.8
C7—C6—C5	110.30 (11)	C13—C14—C9	120.94 (12)
C7—C6—H6A	109.6	C13—C14—H14	119.5
C5—C6—H6A	109.6	C9—C14—H14	119.5
			
O1—N1—C1—C2	−128.32 (12)	C6—C7—C8—C3	56.07 (15)
O2—N1—C1—C2	52.56 (14)	C4—C3—C8—C7	−56.14 (13)
N1—C1—C2—C9	61.75 (12)	C2—C3—C8—C7	178.44 (10)
N1—C1—C2—C3	−173.91 (9)	C1—C2—C9—C14	−122.22 (12)
C9—C2—C3—C4	−176.37 (10)	C3—C2—C9—C14	113.75 (13)
C1—C2—C3—C4	59.53 (13)	C1—C2—C9—C10	60.49 (14)
C9—C2—C3—C8	−56.26 (13)	C3—C2—C9—C10	−63.53 (15)
C1—C2—C3—C8	179.64 (9)	C14—C9—C10—C11	−1.04 (19)
C2—C3—C4—O3	10.01 (17)	C2—C9—C10—C11	176.33 (11)
C8—C3—C4—O3	−113.44 (13)	C9—C10—C11—C12	−0.4 (2)
C2—C3—C4—C5	−176.03 (10)	C10—C11—C12—C13	1.2 (2)
C8—C3—C4—C5	60.52 (13)	C11—C12—C13—C14	−0.6 (2)
O3—C4—C5—C6	112.56 (13)	C12—C13—C14—C9	−0.9 (2)
C3—C4—C5—C6	−61.43 (14)	C10—C9—C14—C13	1.68 (19)
C4—C5—C6—C7	55.04 (14)	C2—C9—C14—C13	−175.70 (12)
C5—C6—C7—C8	−53.99 (14)		

### Hydrogen-bond geometry (Å, °)

**Table:**

*D*—H···*A*	*D*—H	H···*A*	*D*···*A*	*D*—H···*A*
C1—H1B···O2^i^	0.99	2.57	3.4403 (14)	146
C5—H5A···O2^ii^	0.99	2.47	3.4312 (16)	165
C8—H8A···O1^iii^	0.99	2.53	3.3536 (16)	140
C10—H10···O2^i^	0.95	2.50	3.4289 (15)	165

Symmetry codes: (i) *x*, −*y*+1/2, *z*−1/2; (ii) −*x*+1, *y*−1/2, −*z*+1/2; (iii) *x*, *y*−1, *z*.
